# Association between operative technique and intrusive thoughts on health‐related Quality of Life 3 years after APE/ELAPE for rectal cancer: results from a national Swedish cohort with comparison with normative Swedish data

**DOI:** 10.1002/cam4.1402

**Published:** 2018-04-17

**Authors:** Mattias Prytz, Anna Ledebo, Eva Angenete, David Bock, Eva Haglind

**Affiliations:** ^1^ Department of Surgery Institute of Clinical Sciences SSORG ‐ Scandinavian Surgical Outcomes Research Group Sahlgrenska Academy Sahlgrenska University Hospital/Östra University of Gothenburg Gothenburg Sweden; ^2^ Department of Surgery NU‐hospital Organization Trollhättan Sweden

**Keywords:** APE, ELAPE, Intrusive thoughts, Quality of Life, Rectal cancer

## Abstract

The aim of this prospective registry‐based population study was to investigate the association between QoL 3 years after surgery for rectal cancer and intrusive thoughts and to assess the association with the type of surgery (i.e., APE or ELAPE) in a population‐based national cohort. ELAPE has been proposed as a superior surgical technique for distal rectal cancer, but long‐term effects on QoL are not known. There are also no studies on the association of negative intrusive thoughts on patients′ self‐reported Quality of Life following surgery for distal rectal cancer. Negative intrusive thoughts are regarded as a marker of incomplete cognitive processing of the psychological trauma caused by, for example, a cancer diagnosis. Intrusive thoughts have been recognized as an important factor associated Quality of Life outcome following surgery for other malignancies. All Swedish patients operated with any kind of abdominoperineal resection in the years 2007–2009 were identified through the Swedish ColoRectal Cancer Registry (SCRCR)—the APER population. All patients alive 3 years after surgery and willing to participate were included. Data were collected from three different sources: the registry, the original operative notes, and a study‐specific questionnaire regarding health‐related QoL answered by the patients. Questions on QoL from a normative reference population were also collected for comparison. Fifty‐six percent of the APER population reported a low overall Quality of Life. There was no significant difference between the sexes. Among men, there was a difference in overall QoL, with a higher level in the normative population (48%) compared with the male APER population (39%). Overall QoL was compared to a normative Swedish population. Almost half of the patients experienced negative intrusive thoughts, which was associated with a lower overall Quality of Life. The frequency and severity of negative intrusive thoughts were significantly associated with a low overall QoL. There was no difference in overall QoL after standard, compared with extralevator abdominoperineal excision. A large proportion of survivors after abdominoperineal excision for rectal cancer has a Quality of Life compared with a normative population, but many suffer from negative intrusive thoughts, a symptom of stress, which significantly decrease overall Quality of Life.

## Introduction

In recent years, a lot of research has focused on improving the outcome of distal rectal carcinoma treatment. Several studies have shown that the abdominoperineal excision (APE), the procedure indicated when an anterior resection is not possible or feasible to perform, results in inferior oncological outcomes compared with anterior resection (AR)^1^, ^2^. An adjusted version of the standard APE (Extralevator APE ‐ ELAPE) has been proposed to address this 3. Initial short‐term results from case series on ELAPE were promising 4, 5 with surrogate variables suggesting a superior oncological outcome. More recently large population‐based studies 6, 7 on local recurrence and long‐term outcome, including one by our group on a national Swedish cohort 8, have failed to confirm the early suggestion of superior oncological results after ELAPE compared with standard APE 4, 5. The effect of the standard APE procedure and the ELAPE technique on perineal wound healing and on symptoms related to impaired perineal wound healing have been reported 9 previously. It was found that any kind of APE results in a high frequency of perineal symptoms and that there is an association between the ELAPE technique and prolonged perineal wound healing. Prolonged wound healing has also been shown to be associated with increased perineal symptom intensity. This study was undertaken to further investigate the effect of both standard APE and ELAPE on patients′ self‐reported Quality of Life. Quality of Life (QoL) is a broad concept with no clear definition. There is, however, considerable agreement that Quality of Life is a multidimensional experience. One way of addressing this is by categorizing QoL in five dimensions: physical well‐being, material well‐being, social well‐being, emotional well‐being, and development and activity 10.

The last four decades have seen an increased interest in QoL findings related to health research, and the term health‐related Quality of Life (HRQL) has been used. HRQL data are based directly on the patients’ subjective reports of symptoms and functional outcome collected with different kinds of questionnaires. There is an abundance of validated questionnaires for different medical conditions and treatments 11, 12, 13.

Negative intrusive thoughts (NIT) are involuntary unwelcome thoughts that appear suddenly and repeatedly. Negative intrusive thoughts are part of post‐traumatic stress disorder and have been regarded as a marker of incomplete cognitive processing of the psychological trauma caused by, for example, a cancer diagnosis. Intrusive thoughts have been recognized as an important factor associated with Quality of Life outcome following surgery for other malignancies, that is, prostate and breast cancer 14, 15.

The primary objective of this study was to investigate the association between QoL 3 years after surgery for rectal cancer and intrusive thoughts and to assess the association with the type of surgery (i.e., APE or ELAPE) in a population‐based cohort.

The secondary objective was to compare outcome in this cohort with normative data from a large Swedish cohort.

## Method

The design of the study, the population cohort, and the collection of data have previously been described in detail 9, 16. Patients operated with any kind of abdominoperineal resection in Sweden in the years 2007–2009 were identified through the Swedish ColoRectal Cancer Registry (SCRCR). Data were collected from three different sources: the registry, the original operative notes, and a study‐specific questionnaire regarding health‐related QoL answered by the patients. All Swedish hospitals report to SCRCR, and the registry has a coverage of about 97% and good internal validity 17, 18, 19.

Data such as sex, age, body mass index (BMI), American Association of Anaesthesiology (ASA) grade, tumor height, neoadjuvant treatment, open/laparoscopic procedure, pathological T‐stage and N‐stage, and circumferential resection margin were retrieved from the registry. Data on marital status, education, and occupation were collected from the questionnaire (see below).

Descriptions of the perineal part of the dissection (APE or ELAPE, perineal reconstruction, coccyx resection) were not included in the registry at that time. Therefore, operative notes for each patient were collected from the hospital where the patient was operated on to ascertain which technique had been used (i.e., standard APE or ELAPE).

**Figure 1:**
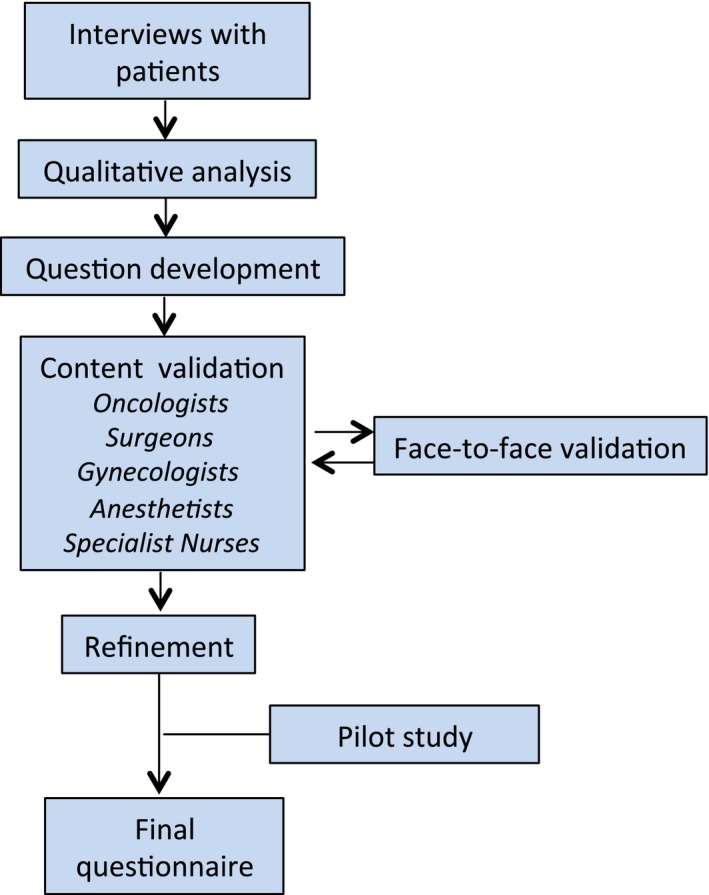
Development of a study‐specific questionnaire.

Three years after rectal surgery the patients completed a specific questionnaire regarding health‐related QoL. The questionnaire was developed according to an established method that involves interviews with patients operated by abdominoperineal excision followed by qualitative content analysis, development, and selection of questions by an expert panel and repeated face‐to‐face validations and subsequent revisions (Fig. 1). This method for development and validation of questionnaires has been described in detail elsewhere 20, 21, 22. The questionnaire covered areas such as socioeconomy, coexisting illness, symptoms, and functional problems. Part of the questionnaire has been used before in studies of patients with prostate and gynecological cancer, respectively 14, 23.

The 1319 rectal cancer patients registered in the SCRCR were cross‐checked with the Swedish Civil Registry in order to avoid contact errors, and an introductory letter was mailed to the 852 surviving patients followed by a phone call. Of these, 703 patients were eligible for inclusion, and 596 agreed to receive the questionnaire by mail. Two weeks after the mailout, a postcard reminder was sent and if the questionnaire was still not returned, a final phone call was made.

**Figure 2:**
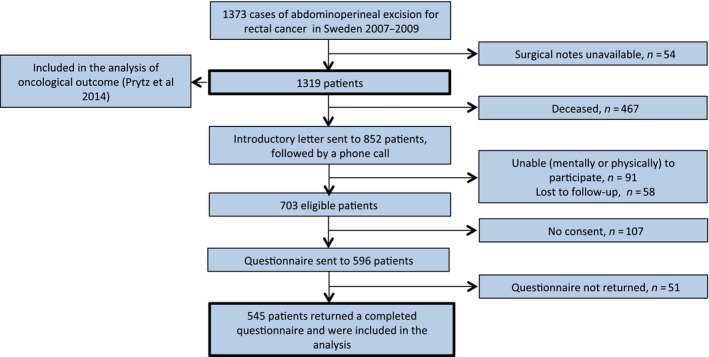
Flowchart of patients.

Five hundred and forty‐five patients returned the questionnaire and were included in the study (Fig. 2).

Overall QoL in the APER population and the normal population was assessed by a 7‐point Likert scale anchored by 0 (no QoL) and 6 (best possible QoL).

The prevalence and extent of intrusive thoughts were assessed by an ordinal scale with seven levels ranging from “Never” to “More than three times per day or all the time”. Answers were dichotomized with a cutoff point between level 2 (Less than once a week) and 3 (At least once a week) prior to analysis.

The questions about the severity of the perceived intrusiveness had five response options: “Irrelevant, I have not had any sudden negative/positive thoughts about the rectal cancer,” “Not at all intrusive,” “A little bit intrusive,” “Moderately intrusive,” and “Very intrusive.” Answers were dichotomized as follows: less than “moderately intrusive” vs. at least “moderately intrusive.”

Comorbidity was interpreted as “Yes” if a patient reported at least one of cardiovascular disease, diabetes, or chronic obstructive pulmonary disease (COPD). Depression was assessed by the question “Are you depressed?” with the answer categories “No,” “Yes,” and “Do not know.” In a earlier study 24, this question and answer combination was reported to result in comparable estimations with a validated instrument if the answers “Yes”/”Do not know” were combined versus the alternative “No.”

American association of anaesthesiology grade: The categories (ASA I‐IV) were collapsed when included in regression analyses. Rectal cancer relapse and the presence of subsequent treatment were dichotomized as Yes/No. Age was entered as a continuous variable in regression analyses and gender as a dichotomous variable.

**Figure 3:**
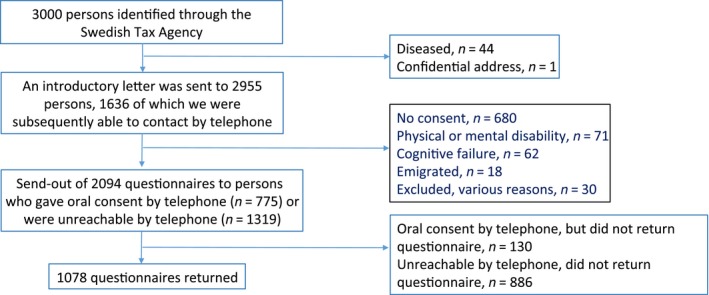
Flowchart of persons included in the population sample. 89% of those who consented to participate returned the questionnaire. The overall response rate was 36%.

A normative reference population of 3000 was collected randomly with the help of the Swedish Tax Agency. An introductory letter was sent to 2955 individuals, 1636 of whom we subsequently managed to contact by telephone. Two thousand and ninety‐four questionnaires were sent out to those who gave verbal consent by telephone (*n* = 775) or who could not be reached by telephone (*n* = 1319). One thousand and seventy‐eight questionnaires were returned and formed the reference population. Eighty‐nine percent of those who agreed to participate returned the questionnaire. The overall response rate was 36% (Fig. 3).

### Statistical analysis

The association between QoL, negative intrusive thoughts, and type of surgery was analyzed with a proportional odds model 25. The proportional odds’ assumption was evaluated, and results are presented as odds ratios with 95% confidence intervals. Potentially influential variables were adjusted for by including them as covariates in the model. The variables were *sex*,* age*,* American Society of Anaesthesiologists classification* (ASA, I‐IV), *tumor stage* (T0–T4), comorbidity (characterized as “Yes” if a patient reported at least one of cardiovascular disease, diabetes, or chronic obstructive pulmonary disease), *marital status* (partner, no partner), and *educational status* (university education, no university education). For sensitivity assessment, results for unadjusted analyses are also presented.

The comparison with the normative data was made using a Cochran–Mantel–Haenszel test of general association 25, stratified by age group (0–49, 50–59, 60–69, 70–79, 80‐years). Separate analyses were made for males and females. Response options were categorized as explained in the tables.

## Results

Of the 1319 patients included in the APER study, 545 answered the QoL questionnaire approximately 3 years after the initial rectal cancer surgery (Fig. 2). The questionnaire response rate was high (91%). A Swedish normative population (*n* = 1078) was used for comparison (Fig. 3).

In the APER population, 60% were male (Table 1). The median age for both men and women was 69 years at the time of filling out the questionnaire. More men lived in a relationship (83 & vs. 59%, *P* = 0.04). There were no differences between the sexes regarding level of education or retirement, and no differences regarding self‐reported comorbidity or depression.

**Table 1:** Demographics for the APER population

	Variables	Female	Male	*P*‐value	All
Age median (Q1; Q3)^a^		68 (62; 75)	69 (63; 76)	0.3122	69 (63; 76)
Sex (%)^a^		218 (40)	327 (60)	0.034	545
ASA grade (%)^a^	ASA 1	67 (30.73)	77 (23.55)	0.2225	144 (26.42)
	ASA 2	123 (56.42)	191 (58.41)		314 (57.61)
	ASA 3	22 (10.09)	51 (15.6)		73 (13.39)
	ASA 4	1 (0.46)	1 (0.31)		2 (0.37)
	Missing	5 (2.29)	7 (2.14)		12 (2.2)
Type of perineal dissection^b^	APE	38 (17.43)	33 (10.09)	0.0393	71 (13.03)
	ELAPE	87 (39.91)	135 (41.28)		222 (40.73)
	Not stated	93 (42.66)	159 (48.62)		252 (46.24)
Neoadjuvant radiotherapy^a^	Yes	194 (88.99)	290 (88.69)	0.9117	484 (88.81)
	No	24 (11.01)	37 (11.31)		61 (11.19)
Tumor stage^a^	T0	12 (5.5)	10 (3.06)	0.0057	22 (4.04)
	T1	15 (6.88)	33 (10.09)		48 (8.81)
	T2	64 (29.36)	122 (37.31)		186 (34.13)
	T3	104 (47.71)	148 (45.26)		252 (46.24)
	T4	19 (8.72)	8 (2.45)		27 (4.95)
	Missing	4 (1.83)	6 (1.83)		10 (1.83)
Lymph node stage^a^	N0	136 (62.39)	208 (63.61)	0.3527	344 (63.12)
	N1	47 (21.56)	83 (25.38)		130 (23.85)
	N2	32 (14.68)	32 (9.79)		64 (11.74)
	NX	3 (1.38)	3 (0.92)		6 (1.1)
	Missing		1 (0.31)		1 (0.18)
Local recurrence^a^	Yes	3 (1.38)	3 (0.92)	0.6151	6 (1.1)
	No	215 (98.62)	324 (99.08)		539 (98.9)
Education^c^	No higher education	189 (86.7)	284 (86.85)	0.8074	473 (86.79)
	University or similar	21 (9.63)	28 (8.56)		49 (8.99)
	Missing	8 (3.67)	15 (4.59)		23 (4.22)
Occupation^c^	Retired	165 (75.69)	251 (76.76)	0.6339	416 (76.33)
	Sick leave	4 (1.83)	8 (2.45)		12 (2.2)
	Unemployed	3 (1.38)	2 (0.61)		5 (0.92)
	Working	46 (21.1)	64 (19.57)		110 (20.18)
	Unknown		2 (0.61)		2 (0.37)
Marital status^c^	In relationship	129 (59.17)	270 (82.57)	<0.0001	399 (73.21)
	Not in relationship	80 (36.7)	50 (15.29)		130 (23.85)
	Missing	9 (4.13)	7 (2.14)		16 (2.94)
Comorbidity^c^	No	116 (53.21)	138 (42.2)	0.0412	254 (46.61)
	Yes	92 (42.2)	171 (52.29)		263 (48.26)
	Missing	10 (4.59)	18 (5.5)		28 (5.14)
Depressed^c^	No	188 (86.24)	274 (83.79)	0.3635	462 (84.77)
	Yes/Don′t know	26 (11.93)	40 (12.23)		66 (12.11)
	Missing	4 (1.83)	13 (3.98)		17 (3.12)

a Retrieved from Swedish Colorectal Cancer Registry.

b Retrieved from operative notes.

c Retrieved from study‐specific questionnaire.

In the normative population, the median age was lower (63 49:72 vs. 69 63:76 years), and the proportion of women in a relationship (72%) was higher than in the APER population (59%) (Table 2). A greater proportion of the normative population had a university education, and more of them were still working compared with the APER population (Tables 1 and 2).

**Table 2:** Demographics for the normative population

	Variables	Female	Male	All
Age Median (Q1; Q3)		61 (46; 71)	64 (53; 74)	63 (49; 72)
Sex		566 (52)	512 (48)	1078 (100)
Education (%)	No higher education	320 (56.54)	343 (66.99)	663 (61.5)
	University or similar	198 (34.98)	147 (28.71)	345 (32.0)
	Missing	48 (8.48)	22 (4.3)	70 (6.49)
Occupation (%)	Retired	260 (45.94)	267 (52.15)	527 (48.8)
	Sick leave	8 (1.41)	2 (0.39)	10 (0.9)
	Unemployed	9 (1.59)	5 (0.98)	14 (1.2)
	Working	276 (48.76)	233 (45.51)	509 (47.2)
	Unknown	13 (2.3)	5 (0.98)	18 (1.7)
Marital status (%)	In relationship	408 (72.08)	413 (80.66)	821 (76.1)
	Not in relationship	154 (27.21)	97 (18.95)	251 (23.2)
	Missing	4 (0.71)	2 (0.39)	6 (0.6)
Depressed (%)	No	474 (83.75)	445 (86.91)	919 (85.0)
	Yes/Don′t know	86 (15.19)	63 (12.3)	149 (13.8)
	Missing	6 (1.06)	4 (0.78)	10 (0.93)
Comorbidity (%)	No	381 (67.31)	320 (62.5)	701 (65.0)
	Yes	185 (32.69)	192 (37.5)	377 (35.0)

Fifty‐six percent of the APER population reported a low overall Quality of Life. There was no significant difference between the sexes (Table 3). Among men, there was a difference in overall QoL, with a higher level in the normative population (48%) compared with the male APER population (39%). The median score for global health‐related QoL (EQ5D VAS) was 80 for both sexes and in both cohorts (not shown in table).

**Table 3:** QoL and intrusive thoughts in the APER and normative population

	Variable	APER Female	Normative Female	APER Male	Normative Male	Comparison Female^a^	Comparison Male^a^	All
Quality of life^b^	High QoL	96 (44.04)	261 (46.11)	127 (38.84)	250 (48.83)	0.4138	0.0015	734 (45.17)
	Low QoL	114 (52.29)	298 (52.65)	189 (57.8)	253 (49.41)			854 (52.55)
	Missing	8 (3.67)	7 (1.24)	11 (3.36)	9 (1.76)			37 (2.28)
Negative intrusive thoughts prevalence	Negative Intrusive thoughts	114 (52.29)	383 (67.67)	144 (44.04)	297 (58.01)	0.0022	0.0010	938 (57.72)
	No negative intrusive thoughts	96 (44.04)	181 (31.98)	177 (54.13)	215 (41.99)			669 (41.17)
	Missing	8 (3.67)	2 (0.35)	6 (1.83)				18 (1.11)
Negative intrusive thoughts frequency^c^	At least 1/week	41 (18.81)	161 (28.45)	48 (14.68)	109 (21.29)	0.0085	0.0039	359 (22.09)
	Less than 1/week	73 (33.49)	222 (39.22)	96 (29.36)	188 (36.72)			579 (35.63)
	Never	96 (44.04)	181 (31.98)	177 (54.13)	215 (41.99)			669 (41.17)
	Missing	8 (3.67)	2 (0.35)	6 (1.83)				18 (1.11)
Negative intrusive thoughts severity^d^	At least “moderately intrusive”	18 (8.26)	52 (9.19)	29 (8.87)	30 (5.86)	0.0456	0.0113	129 (7.94)
	Never had	94 (43.12)	186 (32.86)	163 (49.85)	236 (46.09)			679 (41.78)
	Not or little intrusive	99 (45.41)	323 (57.07)	127 (38.84)	242 (47.27)			791 (48.68)
	Missing	7 (3.21)	5 (0.88)	8 (2.45)	4 (0.78)			26 (1.6)
Positive intrusive thoughts prevalence	Intrusive thoughts	57 (26.15)	440 (77.74)	85 (25.99)	376 (73.44)	<0.0001	<0.0001	958 (58.95)
	No intrusive thoughts	150 (68.81)	121 (21.38)	233 (71.25)	132 (25.78)			636 (39.14)
	Missing	11 (5.05)	5 (0.88)	9 (2.75)	4 (0.78)			31 (1.91)

a *P*‐value for Cochran–Mantel–Haenszel test of general association.

b Dichotomized with a cutoff point between 4 and 5 for the analyses.

c Dichotomized with a cutoff point between level 2 (less than once a week) and 3 (at least once a week).

d Dichotomized: less than “moderately intrusive” versus at least “moderately intrusive”.

The APER group of patients was surgically treated by either abdominoperineal excision (APE) or extralevator abdominoperineal excision (ELAPE) technique (Table 1). This classification could not be made for almost 50% of the cohort, as it was “not stated” in the operative notes.

Negative intrusive thoughts were reported by 52% of women and by 44% of men (*P* = 0.04). Nineteen and fifteen percent of the females and males, respectively, reported such thoughts at least once per week 3 years after their surgery for rectal cancer. Nine percent regarded the severity of the intrusive thoughts as “Moderately intrusive” or “Very intrusive” with no difference between the sexes. Twenty‐six percent of the APER group (Table 3) reported positive intrusive thoughts with no difference between the sexes.

After 3 years, there was no difference in overall QoL after standard, compared with extralevator abdominoperineal excision (Table 4). The frequency and severity of negative intrusive thoughts, however, were significantly associated with a low overall QoL. This association was less certain in regard to positive intrusive thoughts.

**Table 4:** Odds ratios for association between surgical method and intrusive thoughts on overall QoL in the APER population

Variables	Comparison	OR (95% CI)^a^	
		Adjusted^b^	Unadjusted
Surgical method	ELAPE vs. APE	0.91 (0.54; 1.53)	1.04 (0.64; 1.68)
	“Not stated” vs. APE	0.71 (0.42; 1.21)	0.88 (0.54; 1.41)
Negative Intrusive Thoughts (NIT) Prevalence	NIT vs. No NIT	2.61 (1.84; 3.70)	2.30 (1.68; 3.15)
Frequency of NIT	At least once/week vs. Never	6.64 (4.01; 10.99)	5.12 (3.25; 8.05)
	Less than once/week vs. Never	1.80 (1.23; 2.60)	1.64 (1.16; 2.33)
Severity of NIT	“Moderately” or “very” NIT vs. “Never had”	10.09 (5.27; 19.31)	8.64 (4.78; 15.61)
	“Not at all” or “a little bit” NIT vs. “Never had”	2.02 (1.40; 2.90)	1.84 (1.33; 2.55)
Positive Intrusive Thoughts (PIT) Prevalence	PIT vs. No PIT	0.64 (0.44; 0.94)	0.70 (0.49; 0.99)

a Odds ratio for scoring in the lower categories was 0 (no QoL) and 6 (best possible QoL).

b Adjusted for: Sex, age, American Society of Anaesthesiologists classification (ASA, I‐IV), tumor stage (T0–T4), comorbidity (characterized as “Yes” if a patient reports at least one of cardiovascular disease, diabetes, or chronic obstructive pulmonary disease (COPD). Depression was defined as not present if the question “Are you depressed” was answered “No”, marital status (partner, no partner) and educational status (university education, no university education).

Self‐reported depression was associated with negative intrusive thoughts, OR: (95% CI) 3.61 (1.99; 6.56).

## Discussion

In this national cohort of Swedish patients operated with abdominoperineal excision for rectal cancer 3 years earlier, overall QoL was compared to a normative Swedish population.

Almost half of the patients experienced negative intrusive thoughts, which was associated with a lower overall Quality of Life. There seems to be a dose–response relationship between the frequency and intensity of intrusive thoughts and overall Quality of Life, which indicate a causal connection.

The men in the APER population experienced a lower overall QoL than men in the normative population. The cause of this is unknown; it is probably multifactorial and will be subject of further analyses.

The strengths of this study include the study design; it is a recent, large, population‐based cohort, initially including all Swedish patients operated with APE for rectal cancer, during a three‐year period. The questionnaire was specifically designed and validated for patients who had undergone APE. The questions used in the current study have been analyzed previously in a group of patients with prostate cancer 14, 26.

In an earlier analysis of this population, we have reported that perineal morbidity was frequent 3 years after surgery and that such symptom was associated with a lower global health‐related Quality of Life 9.

There are some weaknesses of the study that needs discussing; in 46% of the patients, it was not possible to interpret from the operating notes what kind of perineal dissection had been performed (i.e., standard APE or ELAPE), this of course limits the possibility to specifically relate the QoL to different operating techniques but rather the entire group of patients that has undergone any kind of APE. We regard these results as representative of the APE procedure and the rectal cancer disease and not specific for the used surgical technique; that is, standard APE or ELAPE. Also the differences in age and educational status between the study population and the normative population need commenting; the study population was collected from the registry as it was and the normative population was not designed to match the study population with regard to demographic variables. The normative population was randomly collected persons between the age of 30 and 89. The population was collected with help of the Swedish Tax agency to represent a normal Swedish population and not specifically selected to be a normative control group for this study; hence the difference in age and education between the groups. Accordingly, this population is not entirely compared to the study population with regard to demographic data and in that aspect of course limited in a direct comparison.

Using the global health‐related QoL (a generic instrument) we found no differences in the APER patients compared with the normative population. A Swedish EQ5D survey reported levels of health‐related QoL similar to ours 27. Using our disease‐specific validated, detailed questionnaire, differences in overall QoL were detected, and the design of the questionnaire allows further investigation of possible explanatory variables such as intrusive thoughts. The low prevalence of missing answers is a strength.

The questionnaire was sent out 3 years after the initial surgery, when patients with recurrent disease or severe comorbidity were no longer alive, limiting the study to long‐term survivors. This can also be regarded as a strength as data from this population represents the remaining, long‐term results of oncologically successful rectal cancer surgery 28. Patients with cognitive disorders could not participate.

The lack of baseline data can be regarded as a limitation, but the questionnaire includes numerous questions regarding socioeconomic status and comorbidities, for example, and allows for epidemiological methodology in the analysis 29, 30.

Negative intrusive thoughts have been described as one of the symptoms of post‐traumatic stress syndrome 31, 32. It has been described previously in cancer patients 14, 15, 33 and might be a sign of insufficiently coping with the diagnosis and treatment ^33^. The association between negative intrusive thoughts and low Quality of Life has been reported in patients with prostate cancer 14 and patients with breast cancer 15. There are interesting reports of encouraging results in small groups of patients treated with expressive writing 34.

We are not aware of negative intrusive thoughts having been measured in a normative cohort previously. The results in the normative cohort used here are somewhat surprising, with a larger proportion reporting this symptom of stress than in the cohort of survivors of rectal cancer. For the patients with rectal cancer, however, negative intrusive thoughts were strongly associated with low overall Quality of Life, as has been reported earlier 14, 15.

## Conclusion

A large proportion of survivors after abdominoperineal excision for rectal cancer have a Quality of Life compared to a normative population, but many suffer from negative intrusive thoughts, a symptom of stress, which significantly decrease overall Quality of Life. This symptom needs to be addressed to improve the Quality of Life for rectal cancer survivors.

## Ethical Approval

The study has been approved by the Ethical Committee in Gothenburg, no 406‐2010.

## Conflict of Interest

None declared.
